# Commentary: Antifibrotics in COVID-19 Lung Disease: Let Us Stay Focused

**DOI:** 10.3389/fmed.2020.625440

**Published:** 2021-01-11

**Authors:** Soroush Seifirad, Lance Alquran

**Affiliations:** Department of Internal Medicine, Hackensack Meridian Health Mountainside Medical Center, Montclair, NJ, United States

**Keywords:** COVID-19, antifibrotics, fibrosis, ARDS, Pirfenidone

## Introduction

We read with a particular interest the article entitled “Antifibrotics in COVID-19 Lung Disease: Let Us Stay Focused” published by Chaudhary et al. in your prestigious journal, where the authors discussed their point of view regarding the application of approved antifibrotics in the treatment of COVID-19 patients (1). Dr. Chaudhary et al. believe that since some post-viral ARDS patients recover with only mild residual pulmonary deficits, interventions to prevent mild abnormalities would be unnecessary. They also argued that progressive fibrosis is not an important characteristic in ARDS related to respiratory infections and viral pneumonias. In contrast to these statements, the authors admit it is too early to reliably define the long-term outcomes in patients recovering from a severe COVID-19 infection.

They opposed the hypothesis of applicability of approved IPF treatments in COVID-19 patients and commented against the study of anti-fibrotic agents such as Pirfenidone, in the treatment of COVID-19 patients. The authors believe that the relationship between COVID-19 and IPF is nonsense, and hence stated that Pirfenidone trials are a waste of resources. Nevertheless, loss of similarity between IPF and COVID-19 as stated by the authors does not necessarily mean that Pirfenidone will not be helpful in the treatment of patients with COVID-19.

## Discussion

As I, Soroush Seifirad, discussed previously in my published article, there are several rational pathways in pathogenesis of COVID-19 that will be addressed by treatment with Pirfenidone (2). Having a deep understanding of the mechanism of action of a potential medication is essential in suggesting a new treatment. We should model both the disease and the drug mechanism of action, irrelevant of their current application, and recommend novel treatments accordingly (3, 4). Indeed, a large number of currently approved medications in medicine have been proposed for other totally irrelevant disorders; among them are Amantadine, Sildenafil, and Carbamazepine (5).

The reason behind suggesting Pirfenidone is not simply the similarity of COVID-19 ARDS/Fibrosis and IPF; but the pathogenesis of COVID-19 disease; it's receptor, and the mechanism of action of Pirfenidone. In line with our hypothesis, a recent *in-silico* drug repurposing study suggested promising modeling results for the combination of Pirfenidone and melatonin as a potential treatment to diminish SARS-CoV-2 infection progression and respiratory distress due to cytokine storm (6). Nevertheless, at the end of the day, only clinical trials are going to determine if this hypothetical treatment would work. A series of clinical trials are already being conducted, among them are a Chinese multicenter clinical trial sponsored by Huilan Zhang (Study Identifier: NCT04282902), an American trial by PureTech on LYT-100 (deupirfenidone) and a Spanish study (NCT04607928).

COVID-19 patients suffer from severe inflammation, cytokine storms, oxidative stress, reactive oxygen species damage, and increased permeability of vascular bed. These mechanisms are responsible for the development of ARDS and multi-organ damage (2, 7–9). We do agree with the respected authors that it is premature to consider fibrosis after COVID-19 lung disease irreversible and similar to IPF. With no doubt, we need more data and follow up studies to understand characteristics of COVID-19 lung disease, its sequels and reversibility of fibrosis (1). Nevertheless, as we will discuss this fact does not necessarily contradict applicability of antifibrotics such as Pirfenidone in the treatment of COVID-19 patients.

The anti-inflammatory characteristics of Pirfenidone have been reported in several published articles (3, 4). Pirfenidone decreases TNF-α secretion and plenty of other inflammatory cytokines (10–13). Pirfenidone also blocks NLRP3 inflammasome activation, it ameliorates lipopolysaccharide induced inflammation and fibrosis (14, 15). Pirfenidone strongly inhibits TGF- β 1-induced fibronectin synthesis (16, 17). It also down-regulates the profibrotic gene expression, and collagen secretion (16, 18). Pirfenidone prevents collagen I fibril formation, and up regulates RGS2 which can potentially lead to amelioration of pulmonary fibrosis (19–22). Pirfenidone also inhibits NADPH dependent lipid peroxidation (23). The antioxidant character of Pirfenidone suggests its capability for the treatment of hyperimmune response (3, 24). Pirfenidone could decrease apoptosis and as a result combat sever viral inflammation, ARDS, and ARDS fibrosis (25, 26). Finally, it has been demonstrated that Pirfenidone inhibits the AT1R/p38 MAPK pathway, decreases angiotensin II, and angiotensin II type 1 receptor, as well as angiotensin-converting enzyme (ACE) expression, which will both protect cells from developing fibrosis (LXR-α), and limit entrance of the COVID-19-SARS virus into cells by decreasing ACE receptors (2, 27, 28).

**Figure 1:**
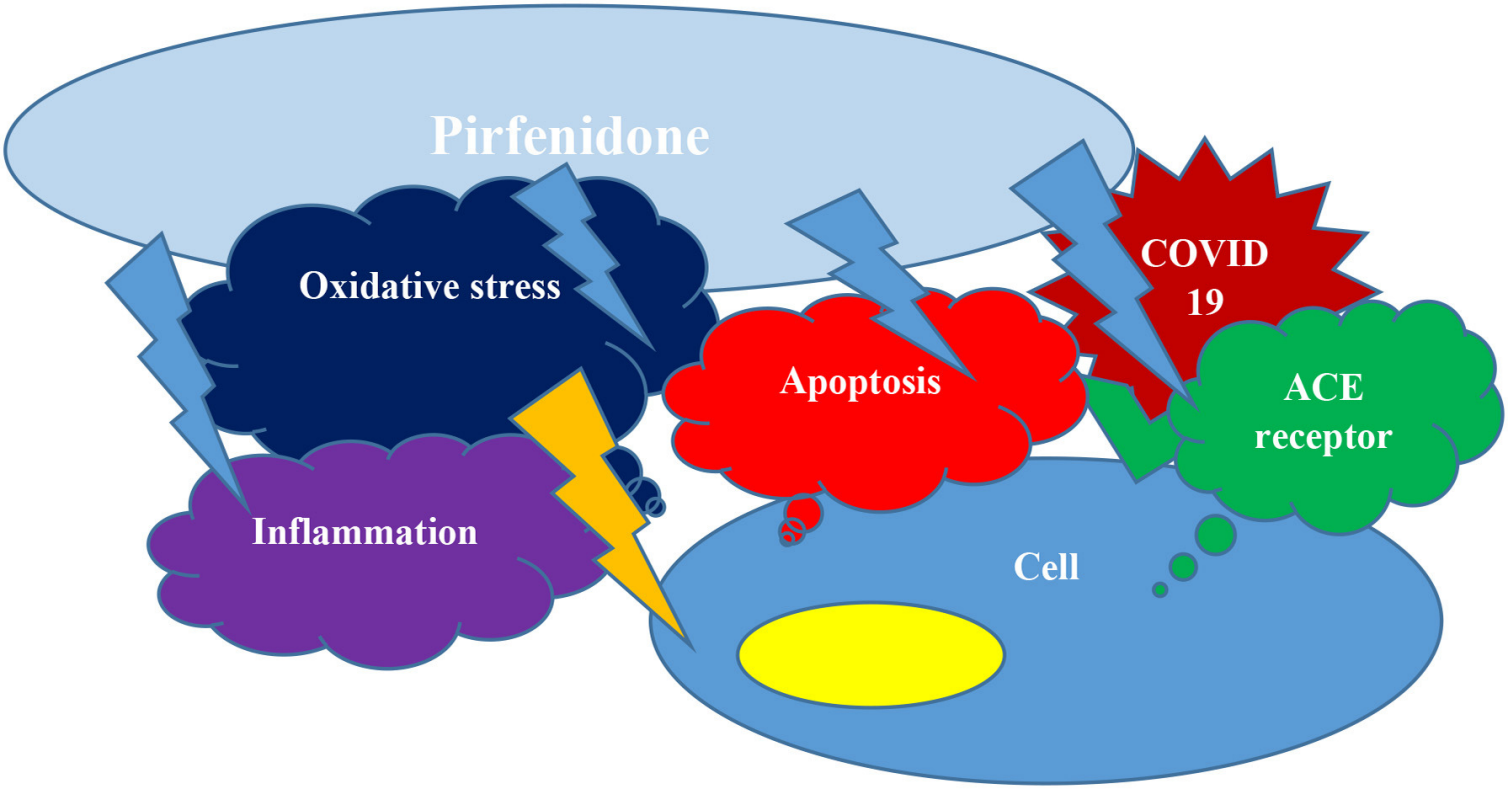
Pirfenidone can hinder apoptosis, may down regulate ACE receptors expression, could decrease inflammation and decrease oxidative stress and hence protect from COVID19 cellular entrance and cytokine storm concurrently.

In conclusion, as we discussed above, Pirfenidone may protect pneumocytes and other cells from COVID-19 invasion and cytokine storm simultaneously by inhibiting apoptosis, downregulating ACE receptors expression, subsiding inflammation by several mechanisms, and improving oxidative stress (Figure 1); (2). We would like to respectfully oppose their statement and support clinical trials of Pirfenidone, particularly for moderate to severe COVID-19 patients.

## Author Contributions

SS: idea and drafting article and final approval. LA: drafting article, English revision, and final approval. All authors contributed to the article and approved the submitted version.

## Conflict of Interest

The authors declare that the research was conducted in the absence of any commercial or financial relationships that could be construed as a potential conflict of interest.
